# How Do Host Plants Mediate the Development and Reproduction of *Phytoseiulus persimilis* (Acari: Phytoseiidae) When Fed on *Tetranychus evansi* or *Tetranychus urticae* Koch (Acari: Tetranychidae)?

**DOI:** 10.3390/insects17020133

**Published:** 2026-01-23

**Authors:** Yannan Zhang, Sijin Bi, Chuqin Huang, Li Ran, Li Yang, Lan Xiao, Qiumei Tan, Endong Wang

**Affiliations:** 1 School of Life Sciences (School of Ecological Forestry), Mianyang Normal University, Mianyang 621006, China; zyn_082@163.com (Y.Z.);; 2 Key Laboratory of Research and Conservation of Biological Diversity in Minshan Mountain of National Park of Giant Pandas, Mianyang Normal University of Sichuan Province, Mianyang 621006, China; 3 Plant Protection Research Institute, Mianyang Academy of Agricultural Sciences, Mianyang 621006, China; 4 Institute of Plant Protection, Chinese Academy of Agricultural Sciences, Beijing 100193, China

**Keywords:** biological control, predatory mites, life table parameter, tertiary trophic relationship

## Abstract

In this study, *P. persimilis* was provided with *T. evansi* and *T. urticae* that had been reared on either bean or potato plants to investigate the effects of both prey and host plant species on the predator’s growth, development, and fitness. The results indicate that the reproductive potential of *P. persimilis* populations fed *T. evansi* was significantly lower than that of populations fed *T. urticae* from the same host plant (*p* < 0.01). *Phytoseiulus persimilis* fed *T. evansi* that had been reared on potatoes showed poorer performance in oviposition period, post-oviposition period, daily egg production, and total egg production compared to those fed *T. evansi* reared on beans (*p* < 0.01). The intrinsic rate of increase (*r_m_*) of *P. persimilis* fed on *T. evansi* reared on potato was 0.08, which was 55.56% lower than that of populations fed on *T. evansi* reared on beans. This study sheds light on the complex interactions among host plants, pests, and their natural enemies, thereby providing a theoretical basis for developing more effective and sustainable management strategies against *T. evansi* that take these intricate ecological relationships into account.

## Simple Summary

*Tetranychus evansi* Baker et Pritchard and *Tetranychus urticae* Koch (Acari: Tetranychidae) are significant agricultural pests. *Phytoseiulus persimilis* (Acari: Phytoseiidae) is one of the most effective natural enemies for controlling *Tetranychus* pests. This study compared the influence of host plants on the biological performance of *P. persimilis* when preying on these two *Tetranychus* species raised on either bean or potato plants. A key finding of this study was that *T. evansi* does not serve as suitable prey for *P. persimilis*. Furthermore, potato plants were shown to indirectly reduce the effectiveness of *P. persimilis* by adversely affecting the pest population itself. This research contributes to a better understanding of the complex interactions among crops, pests, and their natural enemies—insights that are important for developing more effective pest management strategies.

## 1. Introduction

The spider mites *Tetranychus urticae* Koch and *Tetranychus evansi* Baker et Pritchard (Acari: Tetranychidae) are both important pest species within the genus *Tetranychus* Dufor. *Tetranychus evansi* is a highly destructive pest of numerous economically important crops, particularly those in the Family Solanaceae [1,2]. First reported in 1960 from specimens collected on tomato in Mauritius [3], this pest has rapidly spread and demonstrated strong adaptability, becoming a significant invasive agricultural pest worldwide [4,5]. *Tetranychus evansi* poses a considerable threat to the production of solanaceous crops, as well as to a range of other vegetables and ornamental plants in affected regions. *Tetranychus urticae* is a major pest in both open field and protected cultivation systems worldwide [6,7]. It is notorious for its polyphagous nature, having been documented on hosts from more than 140 botanical families [8].

Research interest in the natural enemies of *T. evansi* emerged even before the species was formally described [9]. Early records in the late 1970s noted the co-occurrence of a phytoseiid mite, as well as several predatory insects from the families Miridae, Coccinellidae, and Thripidae, with *T. evansi* populations [10]. This observation prompted wider international efforts to identify effective biological control agents against this pest. In 1982, due to the significant damage caused by *T. evansi* to potatoes and other solanaceous crops in Southern California, the University of California, Riverside launched a research program to evaluate potential natural enemies [11]. In laboratory assays, eight phytoseiid mite species—including *Galendromus annectens* (De Leon), *Galendromus occidentalis* (Nesbitt), *Galendromus porresi* (McMurtry), *Neoseiulus californicus* (McGregor), *Phytoseiulus longipes* Evans, *Phytoseiulus macropilis* (Banks), *Phytoseiulus persimilis* Athias-Henriot, and *Phytoseius hawaiiensis* Prasad—were tested for their predatory efficacy against *T. evansi*. The results showed very low oviposition and survival rates among these predators when feeding on *T. evansi*, indicating their limited suitability as effective biocontrol agents [12].

Phytoseiid mites are among the most widely used commercial biological control agents globally, particularly for managing small arthropod pests such as spider mites [13,14]. Among them, *P. persimilis* is a specialist predator known for its efficacy primarily against *T. urticae* [15,16]. Interestingly, despite the generally poor performance of phytoseiids against *T. evansi* in earlier screenings, de Moraes (1986) found that *P. persimilis* could initially locate and feed on *T. evansi* as readily as on *T. urticae* [17]. A feeding deterrent appeared to prolong the handling time for *T. evansi* eggs, suggesting possible physiological or chemical defenses in this prey species. Despite these findings, the sublethal effects of *T. evansi* on *P. persimilis*—particularly regarding reproduction, development, and population growth—remain poorly understood.

Predator efficacy is shaped by both abiotic (e.g., temperature and humidity) and biotic factors such as prey species, host plant identity, and predator sex [18,19,20,21]. Prey type directly influences predator development, survival, and fecundity [22,23,24,25,26], while host plant traits can mediate tri-trophic interactions, altering pest distribution and natural enemy performance [27,28,29,30,31]. Given these dynamics, this study uses *T. urticae*—a suitable prey for *P. persimilis*—as a control to systematically evaluate the impact of *T. evansi* on the development and reproduction of *P. persimilis*. Additionally, we examine how *T. evansi* reared on different host plants affect the predator’s performance. Our findings help clarify why *P. persimilis* is an inadequate biocontrol agent for *T. evansi* and contribute to the development of integrated management strategies that account for these ecological complexities.

## 2. Materials and Methods

### 2.1. Plants Material and Mite Colonies

Bean (‘Hong Hua’, Shenyang Best Agricultural Technology Co., Ltd., Shenyang, China) and potato (‘Ma ErKe’, Mianyang Green Control Technology Co., Ltd., Mianyang, China) were selected as host plants for rearing *T. urticae* and *T. evansi*. Bean seeds and potato tubers were planted separately in plastic pots measuring 12 cm in diameter. The seedlings were grown using conventional methods until they reached the two-true leaf stage.

The populations of *T. urticae* and *T. evansi* originated from individuals collected in farmland around Mianyang City, Sichuan Province, in 2019. These populations were reared on potted bean seedlings maintained by the College of Life Sciences and Technology, Mianyang Normal University. Prior to this experiment, equal numbers of adult *T. urticae* and *T. evansi* were inoculated onto the two host plant species, and then reared separately for five generations in two chambers located 200 m apart. Fresh host plants were replaced at regular intervals to maintain a stable population of the mites.

Additionally, the colony of *P. persimilis* was supplied by Shoubonong Biotech in 2025. *Phytoseiulus persimilis* populations was maintained in circular plastic containers, each containing a sponge with a height of 5 cm. A layer of filter paper and a sheet of black plastic film, both cut to decreasing diameters, were placed on top of the sponge. Leaves infested with *T. urticae* were then placed on the black plastic film.

### 2.2. Experiment Set-Up

**Figure 1:**
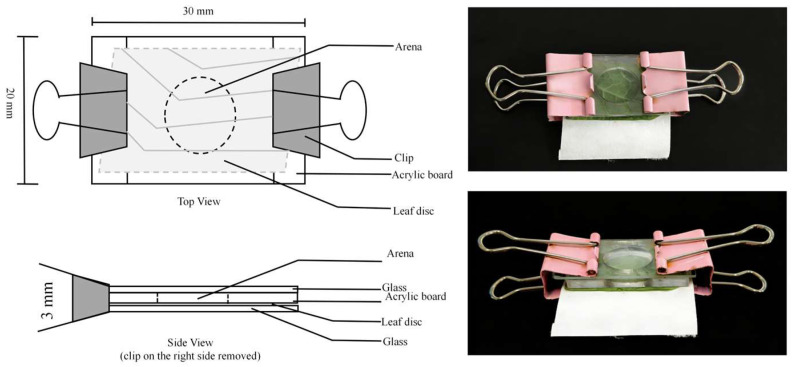
Schematic views and photographs of the experimental unit.

For individual rearing, *P. persimilis* was maintained in custom acrylic chambers. Each chamber was constructed by sealing a layer of host plant leaf, a piece of filter paper, and a glass slide beneath a transparent acrylic board (30 × 20 × 3 mm) with a central opening 10 mm in diameter. The entire assembly was secured at both ends with clips to prevent mites from escaping (Figure 1) [22].

**Figure 2:**
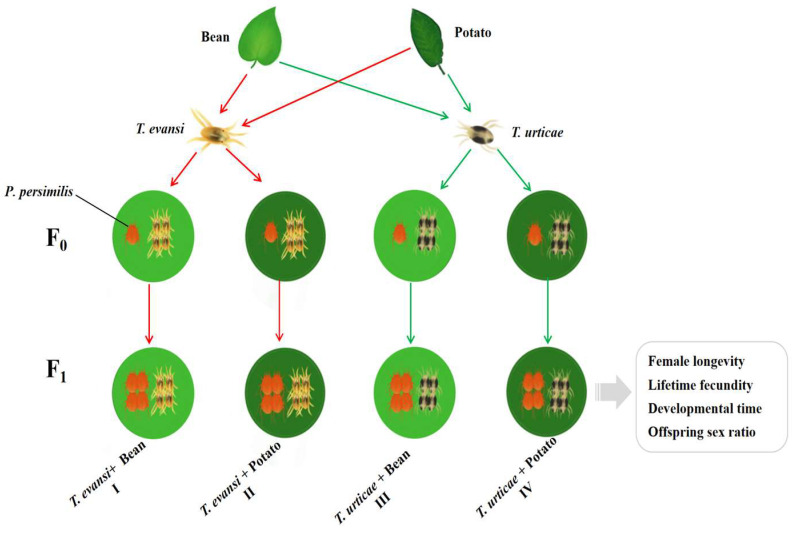
The experimental design (Red arrows indicate *T. evansi;* Green arrows indicate *T. urticae*).

To test the combined effects of prey species and host plant, four treatments were established in which *P. persimilis* were fed: (i) *T. urticae* reared on bean, (ii) *T. urticae* reared on potato, (iii) *T. evansi* reared on bean, and (iv) *T. evansi* reared on potato (see Figure 2 for experimental design). In each treatment, 50 gravid female *P. persimilis* were assigned (five individuals per container, for a total of 10 chambers). For each treatment, eggs were collected from each treatment after a 12 h oviposition period. From the 120 eggs obtained per treatment, three biological replicates of 40 eggs each were prepared. Subsequently, from each of these replicate chambers, the adult female and all but one egg were removed, leaving a single egg per chamber. The developmental stages of each individual *P. persimilis* were recorded twice daily (at 8:00 and 20:00) until they reached adulthood. The duration of each developmental stage was then calculated. During the initial phase (prior to the larval stage), the same leaf disc was kept for each individual. Thereafter, the leaf disc was renewed daily until the experiment concluded. Upon reaching adulthood, each female *P. persimilis* was paired with a male from the same cohort in the rearing chambers. After 24 h, the male was removed, and the female was reared individually until death. Daily, ten adult females each of *T. urticae* and *T. evansi* were provided as prey to *P. persimilis* [29]. The daily egg production of each female was recorded at 24 h intervals until it died, Parameters including the pre-oviposition period, oviposition period, post-oviposition period, longevity, daily fecundity, and cumulative fecundity were then calculated. For each treatment, a minimum of 30 individuals that completed the entire life cycle was required. If any individual escaped or died due to non-experimental causes, the experiment was repeated from the egg stage.

All experiments were conducted under controlled conditions, maintained at: 25 ± 1 °C, 60% relative humidity, and a 16:8 h (light–dark) photoperiod.

### 2.3. Life History Parameters and Statistical Analysis

The life table parameters of the experimental populations were calculated according to the following formulas:

*R*_0_ = ∑*l_x_ m_x_*; (1)

*T* = ∑x *l_x_ m_x_*/*R*_0_ (2)

*r_m_* = ln (*R*_0_)/*T* (3)

*λ* = *e^r_m_^* (4)

*t* = ln (2)/*r_m_* (5)

where *x* represents the age interval in days; *l_x_* denotes the age-specific survival rate of female *P. persimilis*; *m_x_* indicates the age-specific fecundity (average number of female offspring produced per surviving female at age *x*); *R*_0_ is the net reproductive rate (average number of female offspring produced per female over its lifetime); *T* is the mean generation time (the average time interval between the birth of a parent and the birth of its offspring); λ is the finite rate of increase (population multiplication factor unit time); *t* is the population doubling time and *e* is the base of natural logarithms [32].

Two-way ANOVA was used to analyze the effects of plant and prey on the life table parameters of *P. persimilis*. Separately, the Mann–Whitney U test was used to compare the effects of host plant and prey species on specific life history traits: developmental time, longevity, fecundity and oviposition time. All Statistical analyses were performed using SPSS (version 22.0) software. Figures were generated using GraphPad Prism (version 8.0.2).

## 3. Results

### 3.1. Influence of Host Plants on Immature Stages of P. persimilis Fed with T. urticae and T. evansi

**Figure 3:**
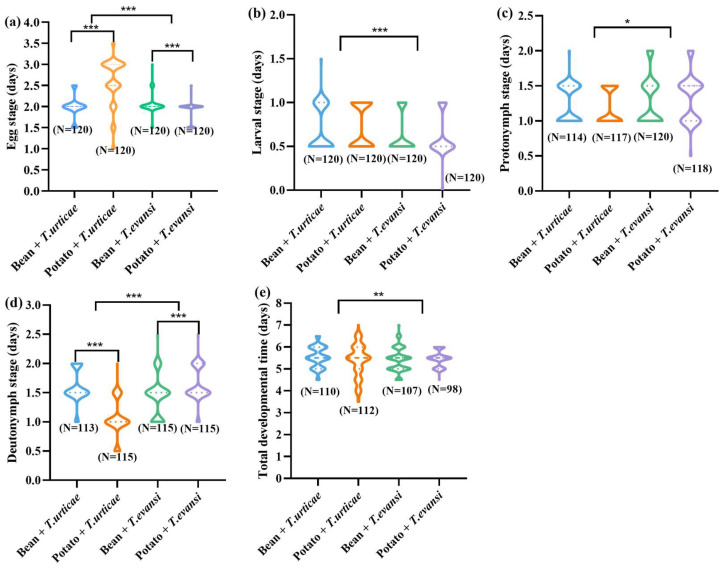
The effect of the host plants on the duration of the immature stages of *P. persimilis* fed with *T. urticae* and *T. evansi* ((**a**) is egg; (**b**) is larva; (**c**) is protonymph; (**d**) is deutonymph; (**e**) is egg to adult). The asterisk stands for significant difference. * *p* < 0.05; ** *p* < 0.01; *** *p* < 0.001.

*Phytoseiulus persimilis* developed into adults after feeding on *T. urticae* reared on beans, *T. urticae* reared on potatoes, *T. evansi* reared on beans, and *T. evansi* reared on potatoes, with 110, 112, 107, and 98 individuals, starting from 120 eggs per treatment (Figure 3). When *P. persimilis* fed on *T. urticae* reared on potatoes, its egg stage was significantly longer than when it fed on *T. urticae* reared on beans (Z = −7.571, *p* < 0.001). The deutonymph development period after feeding on *T. urticae* reared on beans was significantly longer than after feeding on *T. urticae* reared on potatoes (Z = −9.077, *p* < 0.001). When *P. persimilis* fed on *T. evansi* reared on beans, its egg stage was significantly longer than that when fed on *T. evansi* reared on potatoes (Z = −4.872, *p* < 0.001). In contrast, the deutonymphal development period of *P. persimilis* was significantly longer after feeding on *T. evansi* reared on potatoes than reared on beans (Z = −4.317, *p* < 0.001). No significant differences were detected in the larval stage, protonymphal stage durations, or total development time of *P. persimilis* across the four prey-host plant combinations (*p* > 0.05). When preying on *T. urticae* (reared on bean or potato plants), *P. persimilis* had significantly longer egg, nymphal, and adult stages than when preying on *T. evansi* (*p* < 0.001). However, the protonymphal stage of *P. persimilis* was significantly longer when feeding on *T. evansi* compared to *T. urticae*, regardless of the host plant (*p* < 0.001) (Figure 3).

In summary, neither the plant nor the pest mite species had a significant effect on the immature development of *P. persimilis* (*p* > 0.05) (Table 1). In contrast, the plant × mite species interaction was significant for the egg, larval, and nymphal stages (*p* < 0.001) (Table 1), but not for the total developmental time (*F*_(1, 387)_ = 3.478, *p* = 0.063).

### 3.2. Influence of Host Plants on Life Span of P. persimilis Fed with T. urticae and T. evansi

**Figure 4:**
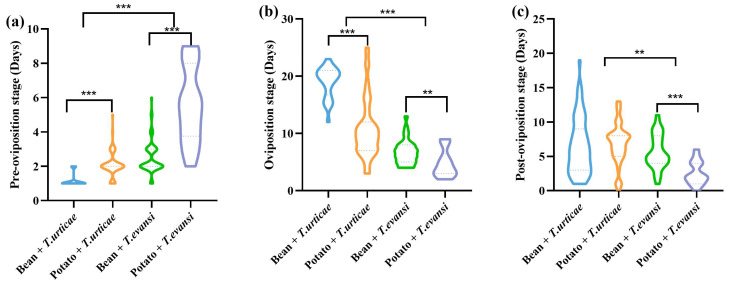
The effect of the host plants on the life span of *P. persimilis* fed with *T. urticae* and *T. evansi* ((**a**) Pre-oviposition. (**b**) Oviposition. (**c**) Post-oviposition). The asterisk stands for significant difference. ** *p* < 0.01; *** *p* < 0.001.

After feeding on *T. evansi* reared on potatoes, the pre-oviposition period of *P. persimilis* was extended by 79.17%, 62.30%, and 55.48% compared to feeding on *T. urticae* reared on beans, *T. urticae* reared on potatoes, and *T. evansi* reared on beans, respectively (Figure 4). The pre-oviposition period of *P. persimilis* was significantly longer when feeding on *T. urticae* reared on potatoes than on beans (Z = −7.492, *p* < 0.001). In contrast, its oviposition period was significantly longer on beans compared to potatoes (Z = −7.652, *p* < 0.001). No significant difference was observed in the post-oviposition period between the two host plant treatments (Z = −1.157, *p* = 0.247). The pre-oviposition period of *P. persimilis* was significantly longer when feeding on potato-reared *T. urticae* than on bean-reared *T. urticae* (Z = −4.995, *p* < 0.001). In contrast, both the oviposition period (Z = −2.919, *p* = 0.004) and post-ovipositions period (Z = −4.024, *p* < 0.001) were shorter on bean-reared prey. Feeding on *T. urticae* (from both beans and potatoes) resulted in a significantly shorter pre-oviposition period for *P. persimilis* compared to feeding on *T. evansi* from the same host plants (Z = −8.059, *p* < 0.001). In contrast, the oviposition period (Z = −9.860, *p* < 0.001) and post-oviposition period (Z = −3.094, *p* = 0.002) were significantly longer when *P. persimilis* preyed on *T. urticae* (Figure 4).

For the adult stage of *P. persimilis*, both host plant species and prey mite species had significant effects (*p* < 0.05). Meanwhile, the host plant × prey mite species interaction was also significant for the pre-oviposition, oviposition, and post-oviposition periods (*p* < 0.05) (Table 1).

### 3.3. Influence of Host Plants on Fecundity of P. persimilis Fed with T. urticae and T. evansi

**Figure 5:**
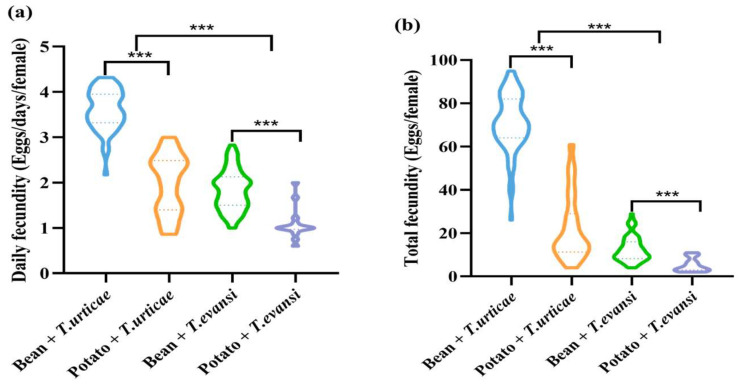
The effect of the host plants on the fecundity of *P. persimilis* fed with *T. urticae* and *T. evansi* ((**a**) Daily fecundity. (**b**) Total fecundity). The asterisk stands for significant difference. *** *p* < 0.001.

For both prey species, potato plants were associated with significantly lower fecundity of *P. persimilis* than with bean plants. When preying on *T. urticae*, daily and total fecundity were lower on potatoes (Z = −9.108, *p* < 0.001; Z = −9.059, *p* < 0.001). Similarly, when preying on *T. evansi*, both fecundity measures were also reduced on potatoes (Z = −4.792, *p* < 0.001; Z = −4.527, *p* < 0.001). Compared to *T. urticae*, consumption of *T. evansi* (from both bean and potato plants) led to significantly lower daily fecundity (Z = −7.923, *p* < 0.001) and total fecundity (Z = −9.266, *p* < 0.001) in *P. persimilis* (Figure 5).

Host plant species, prey pest mite species, and their interaction had significant effects on the daily and total fecundity of *P. persimilis* (*p* < 0.001) (Table 1).

### 3.4. Survival Rate and Daily Female Fecundity of P. persimilis Fed with T. urticae and T. evansi

**Figure 6:**
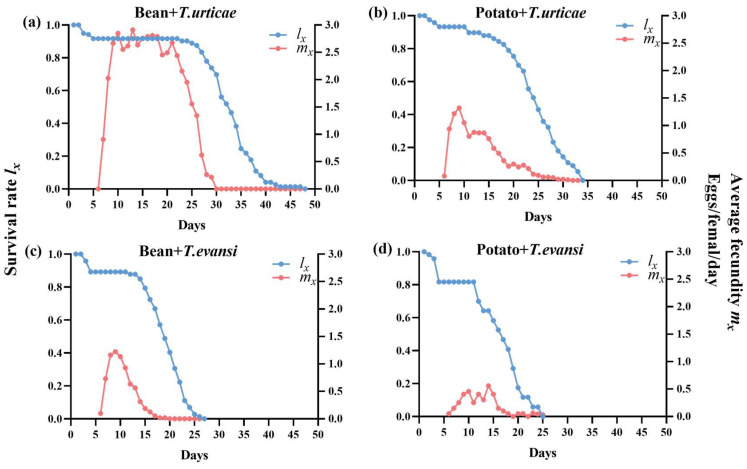
Survival rate (*l_x_*) and age-specific females per day (*m_x_*) of *P. persimilis* fed on *T. urticae* and *T. evansi* reared on two different host plants ((**a**) is *P. persimilis* fed *T. urticae* reared on bean; (**b**) is *P. persimilis* fed *T. urticae* reared on potato; (**c**) is *P. persimilis* fed *T. evansi* reared on bean; (**d**) is *P. persimilis* fed *T. evansi* reared on potato).

As shown in Figure 6, the age-specific survival and fecundity of *P. persimilis* differed by prey and host plant. During development, mortality varied across treatments, with the highest immature mortality observed in the deutonymph stages for *P. persimilis* feeding on *T. evansi* reared on potatoes. In the adult stage, despite most individuals living over 20 days, the onset of mortality differed: it began on day 18 (*T. urticae*/beans), day 6 (*T. urticae*/potatoes), and day 7 for both *T. evansi* treatments, with complete mortality occurring by days 43, 29, 22, and 20, respectively.

In all four treatments, daily fecundity peaked shortly after reproduction began, with maximum age-specific rates reaching 2.91, 1.32, 1.22, and 0.56 eggs per female per day for *P. persimilis* feeding on *T. urticae* reared on beans, *T. urticae* reared on potatoes, *T. evansi* reared on beans, and *T. evansi* reared on potatoes, respectively.

### 3.5. Life Table Parameters of P. persimilis Fed on T. urticae and T. evansi Reared on Two Different Host Plants

The net reproductive rates (*R*_0_) of *P. persimilis* fed on *T. urticae* reared on beans, *T. urticae* reared on potatoes, *T. evansi* reared on beans, and *T. evansi* reared on potatoes were 44.16, 10.30, 6.32 and 2.53, respectively. The intrinsic rates of increase (*r_m_*) for *P. persimilis* across the four treatments were 0.23, 0.19, 0.18, 0.08, respectively. When *P. persimilis* was fed on *T. evansi* reared on potatoes, its intrinsic rate of increase (*r_m_*) was 65.22%, 57.89%, and 55.56% lower than that when preying on *T. urticae* reared on beans, *T. urticae* reared on potatoes, and *T. evansi* reared on beans, respectively (Table 2).

## 4. Discussion

The results of this study indicate that the developmental period from egg to adult for *P. persimilis* when feeding on *T. evansi* reared on potatoes did not significantly differ from that time taken when feeding on *T. urticae* reared on potatoes or beans. However, during the adult stage, parameters including the oviposition period, post-oviposition period, daily egg production, and total egg production all exhibited reduced performance. de Moraes and McMurtry (1986) also found that *P. persimilis* detected and initiated feeding on *T. evansi* as effectively as on *T. urticae* [17]. However, the predation rate of *P. persimilis* was significantly lower on *T. evansi* compared to *T. urticae*. This result suggests that *P. persimilis* does not initially reject feeding on *T. evansi*, but over time, certain compounds in *T. evansi* may gradually impair the reproduction of *P. persimilis*, thereby reducing its effectiveness as a biological control agent against *T. evansi*.

Since *P. persimilis* cannot serve as an effective natural enemy of *T. evansi*, researchers have attempted to identify other viable natural enemies [33]. Sarmento (2004) observed that the amount of fat body in the generalist coccinellid predator *Eriopis connexa* (Germar) (Coleoptera) was significantly lower when it fed on *T. evansi* compared to when it was fed on an aphid species, even though the predator would choose to feed on *T. evansi* when given the option [34]. Another generalist coccinellid predator, *Cycloneda sanguinea*, was found by Oliveira (2005) to be unable to complete its developmental cycle when fed *T. evansi* [35]. These findings collectively indicate that the unsuitability of *T. evansi* as effective prey for natural enemies is not limited to a single predator species.

Several hypotheses have been proposed to explain the unsuitability of *T. evansi* as effective prey for natural enemies. Some researchers have suggested that the dense webbing produced by *T. evansi* may interfere with predation [36], while others have hypothesized that its association with solanaceous host plants could reduce its quality as prey for *P. persimilis* [37]. However, some studies have shown that the main factor leading to reduced fecundity of natural enemy populations is the prey species rather than the host plant. Similar studies have also demonstrated that the development of natural enemies is affected by the prey species they feed on, but not by the plants [38]. However, this study showed that the intrinsic rates of increase (*r_m_*) of *P. persimilis* fed with *T. urticae* reared on beans, *T. urticae* reared on potatoes, *T. evansi* reared on beans, and *T. evansi* reared on potatoes were 0.23, 0.19, 0.18, 0.08, respectively. This suggests that after multiple generations reared on bean-reared *T. evansi*, the reproductive performance of *P. persimilis* approached that of potato-reared *T. urticae*. These results confirm that the host plant of *T. evansi* is a major factor limiting its suitability as prey for *P. persimilis*, consistent with earlier scholarly speculation [39,40].

In tri-trophic interactions, the primary nutrients and the induced secondary metabolites of the host plants influence the physiology and behavior of pests, thereby affecting the growth, development, survival, and reproduction of their natural enemies [41,42]. In this study, *T. evansi* is generally recognized as an oligophagous pest of solanaceous crops. *Tetranychus evansi* has adapted to the secondary metabolites within plants during a long process of co-evolution, while these metabolites cannot be effectively utilized by its natural enemies. It has also been observed in other insects, for instance, Holzinger (1996) found that *Danaus plexippus* (L.) (Lepidoptera: Nymphalidae) can sequester toxic cardenolides from *Digitalis purpurea* L. (Lamiales: Plantaginaceae) through a process of chelation, thereby avoiding predation by its enemies [43]. Fordyce (2001) discovered that *Battus philenor* (L.) (Lepidoptera: Papilionidae) feeds exclusively on *Aristolochia californica* Torr. (Piperales: Aristolochiaceae; presently placed in the genus *Isotrema* Raf.), a plant containing alkaloids, and that the mortality rate and foraging efficiency of *Chrysoperla* Steinmann. (Neuroptera: Chrysopidae) larvae preying on *B. philenor* larvae were significantly lower than those of the control group [44]. Studies have shown that solanaceous crops contain abundant secondary metabolites such as flavonoids, terpenoids, solanine, methyl ketones, and 2-tridecanone [45]. Further investigation is required to determine which specific compounds accumulate in *T. evansi*, thereby inhibiting *P. persimilis* from acting as an effective natural enemy.

Finding effective natural enemies for *T. evansi* is one of the main strategies for controlling this mite. This study demonstrated that *P. persimilis* can effectively control *T. evansi* on beans. However, it remains uncertain whether extending the rearing time of the predatory mite on beans after five generations of *T. evansi* feeding will be more beneficial for the predator’s effectiveness. Athough *P. persimilis* effectively preys on *T. evansi* reared on beans, *T. evansi* itself poses little threat to bean in the field, which presents certain challenges for utilizing predatory mites to control this pest. Therefore, understanding the fundamental reasons behind how the accumulation of solanaceous crop metabolites affects predatory mites—such as their impact on enzyme activity, metabolites, and genes within the natural enemies—can provide insights for identifying effective natural enemies against *T. evansi*.

## 5. Conclusions

This study focused on the effects of prey species and host plants on the growth, development, and fitness of *P. persimilis*. By rearing *T. evansi* and *T. urticae* on beans and potatoes, respectively, and conducting predation experiments, the following core conclusions were drawn: under the same host plant condition, the reproductive potential of *P. persimilis* populations feeding on *T. evansi* was significantly lower than that of populations feeding on *T. urticae* (*p* < 0.01); *Phytoseiulus persimilis* feeding on *T. evansi* reared on potatoes showed the poorest performance in key reproductive indicators such as oviposition period, post-oviposition period, daily egg production, and total egg production (*p* < 0.01). Compared to those feeding on *T. evansi* reared on potatoes, *P. persimilis* feeding on *T. evansi* reared on beans exhibited a 55.56% higher intrinsic rate of increase (*r_m_*). In conclusion, *T. evansi* cannot serve as an effective prey for *P. persimilis*, and this result may be related to the solanaceous crop potato, which indirectly reduces the reproductive potential of *P. persimilis* by affecting *T. evansi*. This study revealed the complex ecological interaction mechanism among host plants, pests, and their natural enemies, thereby providing a theoretical basis for formulating more effective and sustainable management strategies for *T. evansi* that take into account these ecological relationships.

## Figures and Tables

**Table 1:** Effects of Plants and species on the developmental duration, fecundity, and longevity of *P. persimilis*.

Stages	Term	*F* Value	*p* Value
Egg	P	*F*_(1, 387)_ = 1.049	0.306
	S	*F*_(1, 387)_ = 1.049	0.306
	P × S	*F*_(1, 387)_ = 82.679	<0.001 ***
Larval	P	*F*_(1, 387)_ = 3.522	0.061
	S	*F*_(1, 387)_ = 3.523	0.061
	P × S	*F*_(1, 387)_ = 8.112	0.005 **
Protonymph	P	*F*_(1, 387)_ = 1.867	0.173
	S	*F*_(1, 387)_ = 1.868	0.173
	P × S	*F*_(1, 387)_ = 11.358	<0.001 ***
Deutonymph	P	*F*_(1, 387)_ = 0.487	0.485
	S	*F*_(1, 387)_ = 0.488	0.485
	P × S	*F*_(1, 387)_ = 325.171	<0.001 ***
Total developmental time	P	*F*_(1, 387)_ = 0.379	0.538
	S	*F*_(1, 387)_ = 0.380	0.538
	P × S	*F*_(1, 387)_ = 3.478	0.063
Pre-oviposition period	P	*F*_(1, 193)_ = 159.623	<0.001 ***
	S	*F*_(1, 193)_ = 227.256	<0.001 ***
	P × S	*F*_(1, 193)_ = 45.897	<0.001 ***
Oviposition period	P	*F*_(1, 193)_ = 92.111	<0.001 ***
	S	*F*_(1, 193)_ = 284.56	<0.001 ***
	P × S	*F*_(1, 193)_ = 36.395	<0.001 ***
Post-oviposition period	P	*F*_(1, 193)_ = 6.101	0.014 *
	S	*F*_(1, 193)_ = 21.965	<0.001 ***
	P × S	*F*_(1, 193)_ = 9.59	0.002 *
Daily fecundity	P	*F*_(1, 193)_ = 188.23	<0.001 ***
	S	*F*_(1, 193)_ = 236.548	<0.001 ***
	P × S	*F*_(1, 193)_ = 26.896	<0.001 ***
Total fecundity	P	*F*_(1, 193)_ = 199.332	<0.001 ***
	S	*F*_(1, 193)_ = 361.16	<0.001 ***
	P × S	*F*_(1, 193)_ = 107.846	<0.001 ***

Note: P, Plants; S, species; P × S, interaction of plants and species. * *p* < 0.05; ** *p* < 0.01; *** *p* < 0.001.

**Table 2:** Life table parameters of *P. persimilis* fed on *T. urticae* and *T. evansi* reared on two different host plants.

Plants	Net Reproductive Rate (*R*_0_)	Mean Generation Time (*T*, Days)	Intrinsic Rate of Natural Increase (*r_m_*, Day^−1^)	Finite Rate of Increase *λ*	Doubling Time for Population (*t*, Days)
Bean + *T. urticae*	44.16	16.45	0.23	1.26	3.01
Potato + *T. urticae*	10.30	12.44	0.19	1.21	3.70
Bean + *T. evansi*	6.32	10.11	0.18	1.20	3.80
Potato + *T. evansi*	2.53	11.55	0.08	1.08	8.63

## Data Availability

The raw data supporting the conclusions of this article will be made available by the authors on request (If needed, please contact Yannan Zhang at the following email address: zyn_082@163.com).
